# Hedgehog Signaling Pathway Is Active in GBM with GLI1 mRNA Expression Showing a Single Continuous Distribution Rather than Discrete High/Low Clusters

**DOI:** 10.1371/journal.pone.0116390

**Published:** 2015-03-16

**Authors:** Vikas Chandra, Tapojyoti Das, Puneet Gulati, Nidhan K. Biswas, Sarang Rote, Uttara Chatterjee, Samarendra N. Ghosh, Sumit Deb, Suniti K. Saha, Anup K. Chowdhury, Subhashish Ghosh, Charles M. Rudin, Ankur Mukherjee, Analabha Basu, Surajit Dhara

**Affiliations:** 1 National Institute of Biomedical Genomics, P.O. N.S.S. Kalyani, West Bengal 741251, India; 2 Bangur Institute of Neurology, 52/1A, S.N. Pandit Street, Bhawanipore, Kolkata 700098, India; 3 Institute of Post Graduate Medical Education and Research, 244 AJC Bose Road, Kolkata-700020, India; 4 Nil Ratan Sarkar Medical College and Hospital, 138, AJC Bose Road, Kolkata-700014, India; 5 Memorial Sloan-Kettering Cancer Center, 1275 York Avenue, New York, New York 10065, United States of America; University of Navarra, SPAIN

## Abstract

Hedgehog (Hh) signaling pathway is a valid therapeutic target in a wide range of malignancies. We focus here on glioblastoma multiforme (GBM), a lethal malignancy of the central nervous system (CNS). By analyzing RNA-sequencing based transcriptomics data on 149 clinical cases of TCGA-GBM database we show here a strong correlation (r = 0.7) between GLI1 and PTCH1 mRNA expression—as a hallmark of the canonical Hh-pathway activity in this malignancy. GLI1 mRNA expression varied in 3 orders of magnitude among the GBM patients of the same cohort showing a single continuous distribution—unlike the discrete high/low-GLI1 mRNA expressing clusters of medulloblastoma (MB). When compared with MB as a reference, the median GLI1 mRNA expression in GBM appeared 14.8 fold lower than that of the “high-Hh” cluster of MB but 5.6 fold higher than that of the “low-Hh” cluster of MB. Next, we demonstrated statistically significant up- and down-regulation of GLI1 mRNA expressions in GBM patient-derived low-passage neurospheres *in vitro* by sonic hedgehog ligand-enriched conditioned media (shh-CM) and by Hh-inhibitor drug vismodegib respectively. We also showed clinically achievable dose (50 μM) of vismodegib alone to be sufficient to induce apoptosis and cell cycle arrest in these low-passage GBM neurospheres *in vitro*. Vismodegib showed an effect on the neurospheres, both by down-regulating GLI1 mRNA expression and by inducing apoptosis/cell cycle arrest, irrespective of their relative endogenous levels of GLI1 mRNA expression. We conclude from our study that this single continuous distribution pattern of GLI1 mRNA expression technically puts almost all GBM patients in a single group rather than discrete high- or low-clusters in terms of Hh-pathway activity. That is suggestive of therapies with Hh-pathway inhibitor drugs in this malignancy without a need for further stratification of patients on the basis of relative levels of Hh-pathway activity among them.

## Introduction

Hedgehog (Hh) signaling pathway is a developmentally important signaling pathway[1], inactive in normal adult mature cells and found to be aberrantly hyper-activated in a wide range of malignancies [2–5]. Aberrant hyper-activation of this pathway was first identified in Gorlin’s syndrome [6, 7], where an autosomal dominant mutation in the tumor suppressor gene PTCH1 predisposed patients to basal cell carcinoma (BCC) and/or central nervous system (CNS) malignancy medulloblastoma (MB) [8]. PTCH1 is a 12-pass transmembrane (TM) receptor which in absence of its ligand inhibits the other 7-pass TM receptor SMO, keeping it from transmitting the signal. When PTCH1 binds to the soluble ligand sonic hedgehog (SHH)—or inactivated by loss-of-function mutation, as in Gorlin’s syndrome—this inhibition on SMO is withdrawn and the pathway is turned on. PTCH1 has 57% sequence homology and also functional similarity with another TM receptor PTCH2 [9, 10]. The major intracellular players of this pathway in human are STK36, a serine/threonine kinase also known as fused (Fu), suppressor of Fu (SuFu), KIF27, a member of mammalian kinesin family, and finally the signal is transduced by Hh-transcription factors GLI1, GLI2 and GLI3 [11]. GLI1 is the primary representative of the active Hh-pathway. GLI1 and/or GLI2 are up-regulated upon activation of this pathway and further regulate transcriptions of various target genes—for instance, SNAI1 is a bona fide target of GLI1 transcription factor [12]. Role of GLI3 is context dependent, shown to play opposing inhibitory roles [13].

Hh-pathway is aberrantly hyper-activated in 100% of BCC patients [14] and in approximately 30% of MB patients, mostly driven by a loss-of-function mutation in PTCH1[15]. Other than these two, Hh-pathway is ligand-driven in almost all other malignancies studied till date[4]. Ligand-driven Hh-pathway activity is more complex to understand—this activation could be through autocrine and/or paracrine mechanisms [16]. More than 50 registered clinical trials on various cancers are currently going on (https://clinicaltrials.gov, accessed on June 1^st^ 2014) with a series of pharmacological Hh-pathway inhibitors [17]. Systemic administration of Hh-pathway inhibitor Vismodegib (GDC 0449) is already approved by the US Food and Drug Administration (FDA) for the clinical use in BCC[18]. Clinical trial results with MB is also satisfactory [2] [18, 19] but there have been disappointing results in ovarian and colon cancers—malignancies where the pathway is ligand-driven [20, 21]. More detailed investigations are needed to understand the relevance of this pathway in this class of malignancies.

Our disease model here is glioblastoma multiforme (GBM)—a lethal CNS malignancy showing dismal prognosis with standard clinical care of surgery, adjuvant radiotherapy and chemotherapy [22]. Pharmacological inhibition of ligand-driven, aberrantly hyper-activated Hh-pathway is a potential therapeutic approach in this malignancy[3]. Unlike MB, role of this pathway in GBM is less clearly understood. Moreover, some controversies have been raised about the relevance of this pathway in GBM. Dahmane *et al*., in 2001 implicated importance of this pathway in brain development and in CNS tumorigenesis [23]. While Hu *et al*., in 2003 presented evidence of lack of Hh pathway activity in high grade gliomas [24]. However, Bar *et al*., in 2007 showed higher expression of GLI1 in 5 out of 19 GBM cases (26%) and found good correlation with SHH ligand expression in these samples suggesting ligand-driven activity of Hh-pathway [25]. Very recently in 2013, Filbin *et al*., demonstrated synergistic inhibition of PI3K— and Hh—pathway together as a better therapeutic approach in GBM [26]. We analyzed 11 canonical Hh-pathway component genes from the recently published [27] RNA sequencing (RNA-Seq) transcriptomics data on a large clinical sub-cohort (N = 149) of TCGA-GBM database (https://tcga-data.nci.nih.gov/tcga/, accessed on May 20^th^ 2014). We compared Hh-transcription factor GLI1 mRNA expression of GBM with that of MB in order to have a better understanding of the comparative status of Hh-pathway activity in GBM relative to MB as a reference. We demonstrate here ligand-driven up-regulation and vismodegib-driven down-regulation of Hh-pathway activity and induction of apoptosis/cell-cycle arrest with clinically achievable dose of vismodegib to GBM patient-derived neurospheres *in vitro*.

## Materials and Methods

### Patient cohort and collection of tumor biopsy samples

The two ethical committees, institutional review board (IRB) of Bangur Institute of Neurology (BIN) and Review Committee for Protection of Research Risks to Humans (RCPRRH) of National Institute of Biomedical Genomics (NIBMG), both approved this study. Following the guidelines of the Declaration of Helsinki, written consents from all the patients (N = 19) were taken to include them in the study in compliance with IRB approvals of the respective institutes and hospitals. The patient diagnosis were confirmed by radiographic imaging (MRI/CT scan) followed by histopathology as the list of patients is given in the S1 Table. Henceforth, our GBM cohort will be written as NIBMG-GBM. We used GLI1 gene expression data from the MB cases (N = 56) that were already published in NEJM 2009[19] along with a single case of MB from our repository. Also, we downloaded the RNA-Seq based whole transcriptomics data (https://tcga-data.nci.nih.gov/tcga/ accessed on May 20^th^ 2014) and extracted gene expression results of 11 canonical Hh-pathway component genes from the already published large sub-cohort (N = 149) of TCGA-GBM cases [27].

### Neurospheres culture *in vitro*

Single-cell suspensions were prepared from the tumor biopsy samples with the help of enzymatic digestion by Liberase selection grade (Roche Diagnostics GmbH, Mannheim, Germany, cat # 05401046001). Then they were seeded with StemPro NSC SFM media (Invitrogen, Grand Island, NY, USA cat # A10509-01) in tissue culture flasks and incubated for approximately two weeks at 37°C in a humidified chamber with 5% CO_2_ till the appearance of neurospheres.

### Preparation of Shh-enriched conditioned media (Shh-CM)

HEK293-ShhNp cells (a kind gift from Philip Beachy’s laboratory), were grown to ∼ 80% confluency; growth medium was replaced with fresh StemPro NSC SFM medium and incubated for 48 hours. Active ligand Shh-enriched conditioned medium (Shh-CM) was then collected, filtered (pore size, 0.22 μm) and stored at -80°C in small aliquots.

### Shh-CM and vismodegib treatment to neurospheres *in vitro*

Vismodegib (GDC-0449) was purchased from Selleckchem LLC, USA (Cat# S1082) through Pro Lab Marketing Pvt. Ltd., India. Approximately 10^6^ cells/ml/well from the single cell suspensions of GBM neurospheres were seeded in 6-well plates and treated with Shh-CM (50% v/v with growth media) and clinically relevant doses (0μM, 25μM, 50μM and 100μM) of vismodegib for 5 days.

### DNA/RNA extraction and quantitative reverse transcriptase polymerase chain reaction (qRT-PCR)

DNA/RNA was extracted using AllPrep DNA/RNA mini kit (Qiagen GmbH, Germany cat# 80204) and quantified using nanodrop. Reverse transcription was carried out using high capacity cDNA reverse transcription kit (Applied Biosystems, Warrington, UK cat # 4368814). Quantitative measurement of target gene expression relative to GAPDH was performed in triplicates using Power SYBR Green PCR Master Mix (Applied Biosystems, Warrington, UK cat # 4368706) following the manufacturer’s recommendations in an ABI 7900HT Fast real Time PCR system. Relative expression was defined by 2^-ΔCt^, where ΔCt = Ct_target gene_—Ct_GAPDH_. The primer sequences are given in the S2 Table.

### Tracking cell division by CFSE staining following vismodegib treatment

Single cell suspensions of neurospheres were stained with Carboxyfluorescein succinimidyl ester (CFSE) using CellTrace CFSE Cell Proliferation Kit (Life technologies, Oregon, USA cat# C34554) following manufacturer’s protocol. The stained cells were divided into two equal parts, one part treated with DMSO and the other part was treated with 50μM vismodegib for 5 days. CFSE staining intensities were measured by flow cytometry (BD Acuri C6, BD BioSciences, USA) after 5 days of treatment.

### Annexin V staining for apoptosis

Approximately 10^6^ cells /ml were seeded in 6-well plates and treated with either DMSO or 50μM vismodegib consecutively for 5 days. At the end of 5 days the cells were harvested and stained with annexin V. Annexin V staining was done using Alexa Fluor 488 Annexin V/Dead Cell Apoptosis Kit (Life technologies, Oregon, USA cat# V13241) following manufacturer’s protocol.

### Statistical analysis

Pearson’s correlation coefficients (r) were determined on the expression of 11 canonical Hh-pathway component genes in TCGA-GBM cases. Multiple testing correction (MTC) was done with a False Discovery Rate (FDR) of 0.1, to find out significant correlations (cut-off of statistical significance |r| > 0.17). Student’s t-test was used to compare GLI1 mRNA expressions between MB, GBM patients and patient-derived neurospheres. To compare the differences in apoptotic cell numbers (annexin V positive cells) of DMSO- and vismodegib-treated cells, for the *i* ^th^ set of experiments, we translated the number of viable vismodegib-treated cells as percentage of viable DMSO-treated cells (u_*i*_). The mean of u_*i*_ was compared with 100, using univariate t-test. Dose-dependent changes of GLI1 expression in neurospheres were tested by ANOVA followed by multiple comparison tests. All statistical analyses were done using R (http://www.r-project.org) and GraphPad Prism 6 (http://www.graphpad.com).

## Results

### Expression correlations of canonical Hh-pathway component genes in GBM

**Fig 1:**
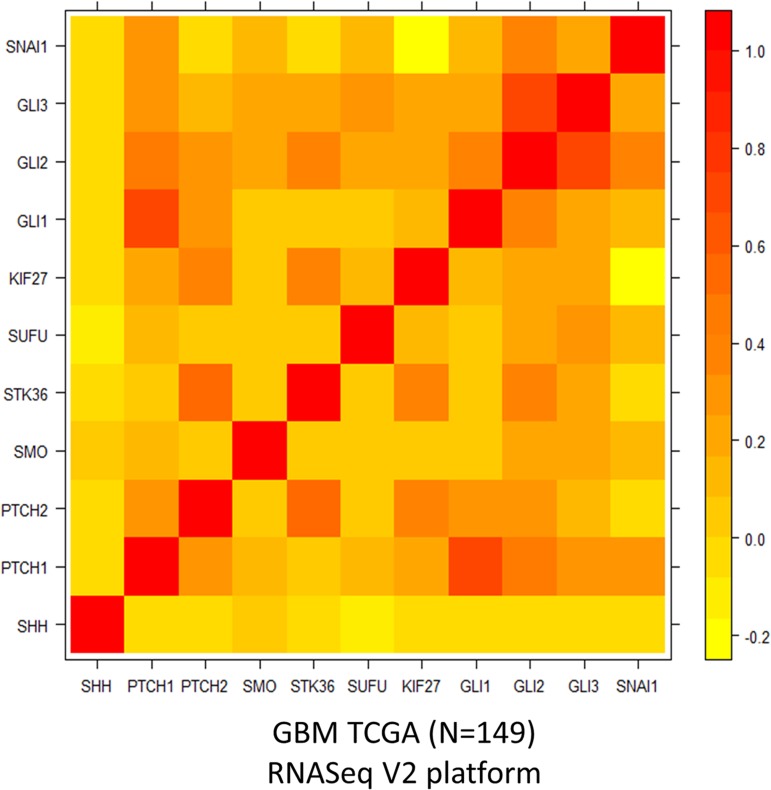
Heat map of correlation matrix for the expression of 11 canonical Hh-pathway component genes in TCGA-GBM (N = 149, significant cut-off |r|>0.17, MTC with FDR 0.1).

A strong correlation (r = 0.7) between GLI1 and PTCH1 mRNA expression was observed among the 149 cases of TCGA-GBM database as shown in Fig. 1. This is a hallmark of Hh-pathway activity [19]. Statistically significant correlations of mRNA expression of GLI1 with that of the other two Hh-transcription factors, GLI2 (r = 0.34) and GLI3 (r = 0.21) were also observed. Other than this, significant correlations (correlations above significance cut-off |r| = 0.17) between intermediate Hh-pathway component gene STK36 with PTCH2 (r = 0.58), KIF27 (r = 0.37), GLI2 (r = 0.34) and GLI3 (r = 0.21) were observed. Hh-pathway downstream target SNAI mRNA expression was significantly correlated with that of PTCH1 (r = 0.29), GLI2 (r = 0.38) and GLI3 (0.24). Cumulatively these correlation patterns are suggestive of activity of the canonical Hh-pathway in this malignancy.

### GLI1 mRNA expression pattern in GBM shows a single continuous distribution rather than discrete high- or low- GLI1-expressing clusters

**Fig 2:**
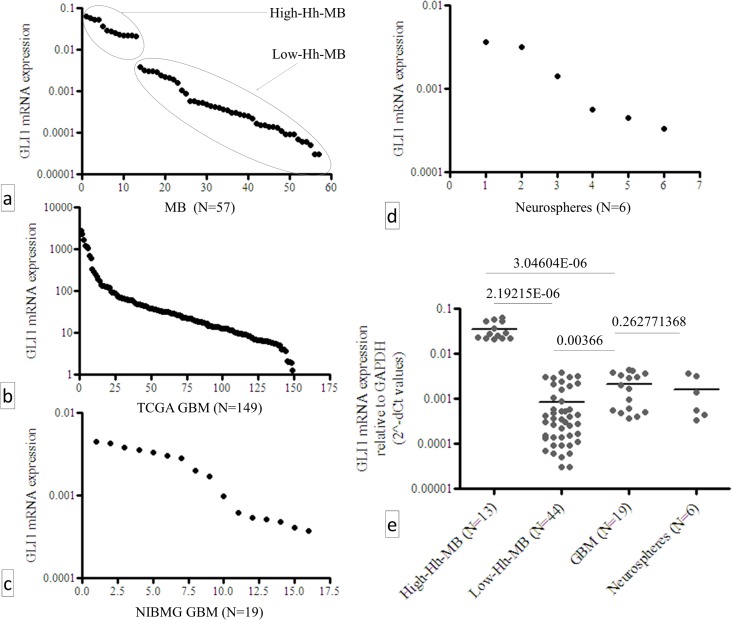
Log scale distribution of GLI1 mRNA expression in a) already published MB cases, along with 1 new case of MB from our repository b) distribution of GLI1 mRNA expression (RNA-Seq data) of the TCGA-GBM sub-cohort (N = 149), c) distribution of GLI1 mRNA expression in NIBMG-GBM cases (N = 19), d) distribution of GLI1 mRNA expression in GBM patient-derived early passage neurospheres (N = 6) and e) comparison of median GLI1 mRNA expression levels of high-Hh-MB (N = 13), low-Hh-MB (N-44), NIBMG-GBM (N = 19) and GBM patient-derived neurospheres (N = 6).

In order to estimate the quantitative levels of GLI1 mRNA expression in GBM—how high is “high” and how low is “low”—we compared GLI1 mRNA expression of NIBMG-GBM clinical cohort (N = 19) with that of an already published clinical cohort of MB (N = 56) [19]. Along with these 56 cases we included a single MB case from our repository in the analysis. Log scale distribution of GLI1 mRNA expression (2^-ΔCt^ values) in MB showed two discrete clusters as high- and low- Hh MB groups as shown in Fig. 2A. The single case of MB from our NIBMG repository was clustered along with the other 12 high-Hh-MB patients. Unlike MB, no such discrete high- or low- Hh clusters in GBM was observed despite the differences between minimum and maximum levels of GLI1 mRNA expression within the same cohort of GBM varying within 3 orders of magnitude (Fig. 2B and C). Similarly, the minimum and maximum GLI1 mRNA expression among the GBM neurospheres also varied in 2 orders of magnitude (Fig. 2D). The median GLI1 mRNA expression of NIBMG-GBM (N = 19) when compared with that of the high-Hh-MB (N = 13) and with the low-Hh-MB (N = 44), it was found to be 14.8 fold lower (p-value 3.04604E-06) than that of the high-Hh-MB but 5.6 fold higher (p-value 0.0037) than that of the low-Hh-MB. There was no significant difference (p-value 0.2627) observed between the median GLI1 levels of NIBMG-GBM patients and patient-derived neurospheres, as shown in Fig. 2E.

### Ligand-driven up-regulation and vismodegib-driven down-regulation of GLI1 mRNA expression in GBM neurospheres *in vitro*

**Fig 3:**
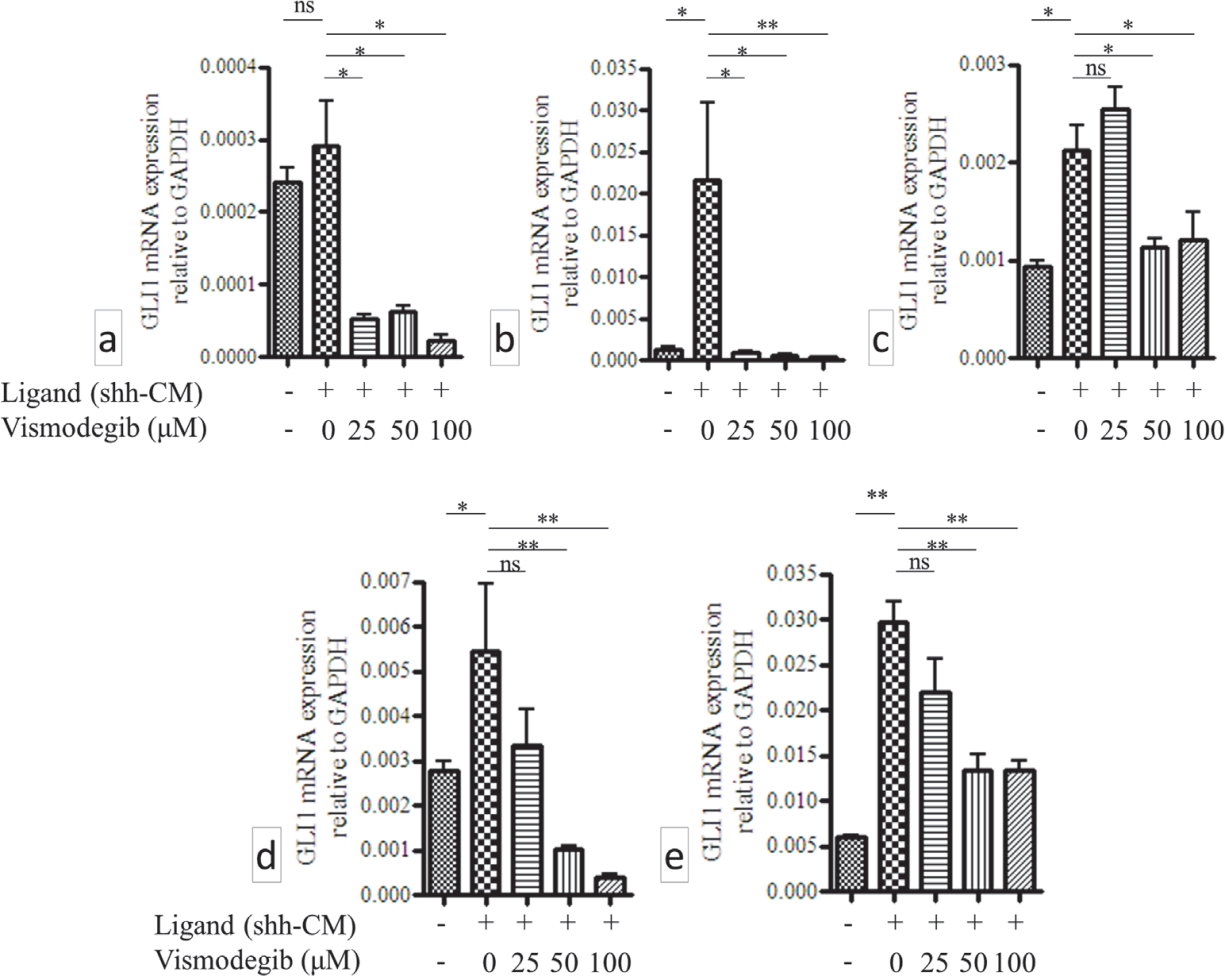
Ligand driven up-regulation and vismodegib driven down-regulation of GLI1 mRNA expressions in 5 GBM neurospheres a) A49910, b), B0027, c) B0043, d) B0051 and e) M45481 (* p-value < 0.05, ** p-value <0.01, *** p-value 0.001 and ns, not significant).

Next, we demonstrated direct up- and down- regulation of Hh-pathway activity in GBM neurospheres *in vitro* (N = 5) by Hh ligand (shh-CM) and clinically relevant doses of vismodegib (25μM, 50μM and 100μM) respectively. As shown in Fig. 3 there was statistically significant up-regulation of GLI1 mRNA expression in 4 out of 5 GBM neurospheres when treated with 50% (v/v) Shh-CM *in vitro*. Significant down-regulation of GLI1 mRNA levels in a dose-dependent manner was observed in all 5 neurospheres at 3 different clinically relevant doses of vismodegib (Fig. 3).

### Vismodegib induces apoptosis and cell cycle arrest in GBM neurospheres *in vitro*

**Fig 4:**
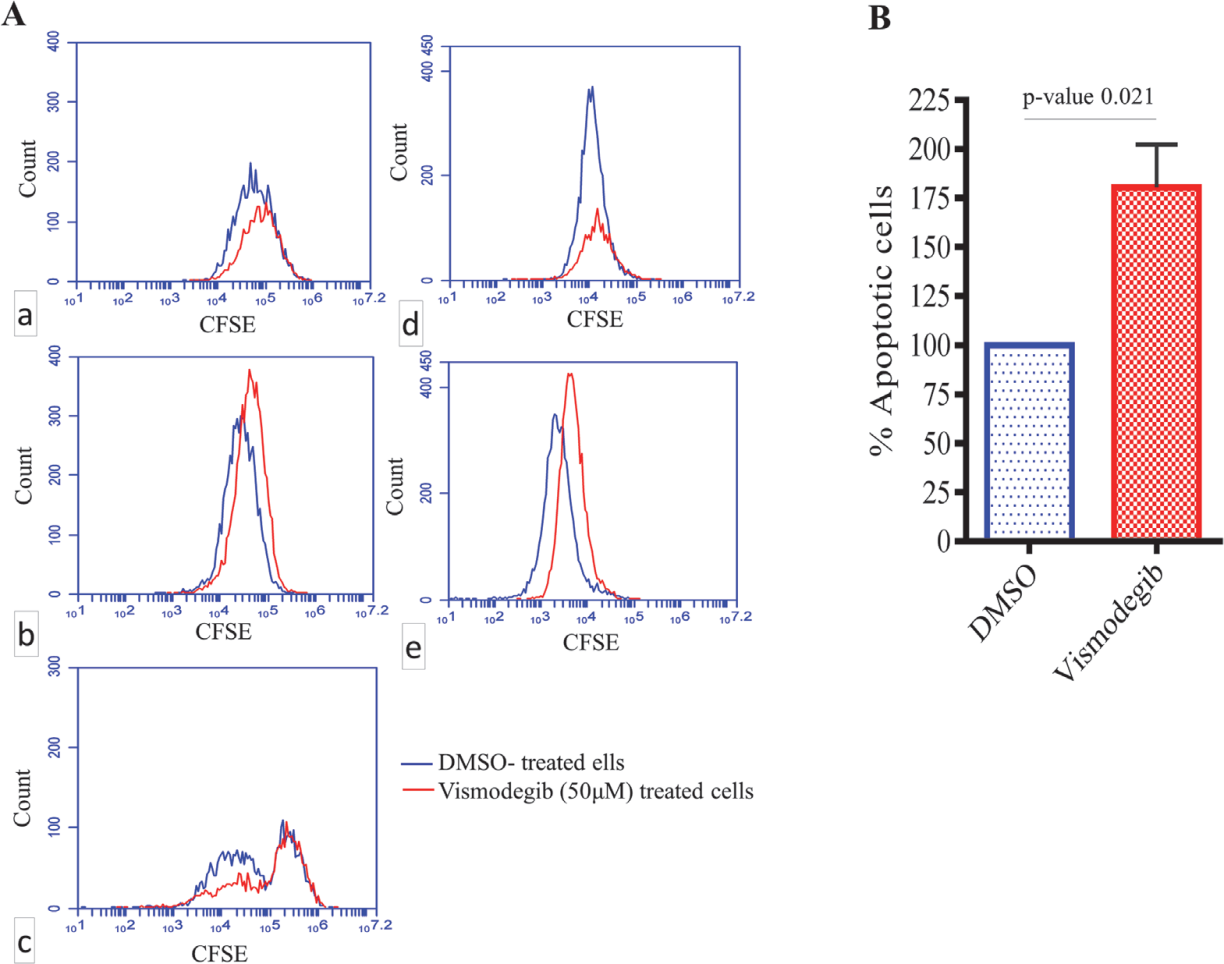
A, cell cycle arrest in GBM neurospheres with vismodegib treatment *in vitro* a) A49910, b), B0027, c) B0043, d) B0051 and e) M45481. **B**, Number of apoptotic cells (annexin V positive) in GBM neurospheres treated either with DMSO or with 50 μM vismodegib.

Finally, we tested if clinically achievable dose (50μM) of vismodegib exerts an effect on cellular proliferation and apoptosis of GBM neurospheres *in vitro* irrespective of their relative endogenous levels of GLI1 mRNA expression. As shown in Fig. 4A B and C, neurospheres with relatively lower endogenous level of GLI1 mRNA expression (as shown in Fig. 3 B) and neurospheres with relatively higher endogenous level of GLI1 mRNA expression both showed cell cycle arrest with vismodegib treatment, as demonstrated by CFSE staining. However, rest of the three neurospheres did not show any growth arrest but a 1.75 fold increase (p-value 0.021) in the average number of apoptotic cells in all of them (N = 5) was observed when treated with similar dose (50 μM) of vismodegib for 5 days *in vitro*. Induction of apoptosis was demonstrated by annexin-V positive cells in vismodegib-treated neurospheres compared to their DMSO-treated controls (Fig. 4B). Altogether, the effect of ligand-driven up-regulation, vismodegib-driven down regulation of GLI1 mRNA expression levels (Fig. 3) and also induction of apoptosis/cell cycle arrest in the GBM patient-derived neurospheres (Fig. 4A and B) were observed irrespective of their relative endogenous levels of GLI1 mRNA expression.

## Discussion

In this paper we revisited the role of Hh-pathway in GBM upon the availability of TCGA-GBM database. First time we demonstrated here a strong expression correlation of GLI1 with PTCH1 expression by utilizing the large clinical cohort of TCGA-GBM database. Strong correlation of GLI1 and PTCH1 expression is a hallmark of Hh-pathway activity [19], since PTCH1 is a bona fide target of GLI1 transcription factor [11], confirming the relevance of this pathway in this malignancy. The previous disagreements on Hh-pathway activity in this malignancy, as presented in the introduction section of this manuscript [24], might have been because of including smaller sample sizes—since the inter- and intra- tumor heterogeneity in GBM could be notorious.

Although GLI1/PTCH1 correlation confirmed the relevance of Hh-pathway in GBM, we sought comparative quantitation of GLI1 mRNA expression in this malignancy—how low is “low” and how high is “high”—compared to another lethal CNS malignancy such as MB [19]. We used MB as a reference because the role of Hh-pathway is more clearly known in this malignancy. Since we used the same set of qRT-PCR primers to estimate GLI1 expression in both MB and NIBMG-GBM, the 2^-ΔCt^ values were comparable. GLI1 mRNA expression of the single MB case from our repository, which was included in the study, clustered along with the other 12 high-Hh-MB cases, substantiating consistency of our method of estimation. Interestingly, we observed median GLI1 mRNA expression in GBM to be significantly lower than that of the high-Hh-MB patients but significantly higher than that of the low-Hh-MB patients. A plausible explanation of this observation is as follows. Hh-pathway activity is driven by a loss-of-function mutation of PTCH1 gene—which is a negative feed-back regulator of Hh-pathway—in case of MB and in BCC [28]. Since the negative feedback regulator of the pathway (PTCH1) is mutated in MB the overall levels of the pathway activity, as estimated by GLI1 expression levels, is un-attenuated and remains higher compared to malignancies where PTCH1 is wild type. Malignancies where PTCH1 is wild type—such as in GBM, pancreatic cancer, ovarian cancers etc.—the resultant effect of ligand-driven aberrant activation of Hh-pathway might be “dampened” or “mellowed down” by PTCH1-mediated negative feed-back mechanisms. However, it is a subject to a far more detailed investigation comparing several malignancies across the board in order to generalize this concept of relative quantitation of Hh-pathway activity between malignancies—particularly between malignancies where the aberrant activity of this pathway is ligand-driven versus it is PTCH1 mutation-driven. Our analysis revealed, unlike MB, GLI1 mRNA expression in GBM to have a single continuous distribution rather than discrete high- or low- Hh expressing clusters. This distribution pattern needs to be taken into account while recruiting GBM patients in clinical trials for therapies with Hh-inhibitors. Technically there is no discrete “cut-off” for high-Hh or low-Hh expressing GBM in order to stratify patients for therapy with Hh inhibitor drugs, despite the inter-tumor differences in GLI1 expression among the patients varied in 3 orders of magnitude. Similar pattern was observed in GBM patient-derived neurospheres as well. Therefore, we sought to determine if there was a direct effect of Hh inhibition by vismodegib (GDC-0449), a pharmacological inhibitor of Hh-pathway and a clinically approved anticancer drug [18], on these GBM neurospheres *in vitro* irrespective of their relative endogenous levels of GLI1 mRNA expression. We clearly demonstrated statistically significant ligand-driven up-regulation of GLI1 mRNA expression and statistically significant vismodegib-driven down-regulation of GLI1 mRNA expression by 3 clinically relevant doses of vismodegib in a dose dependent manner in GBM neurospheres *in vitro*. Also, we demonstrated induction of apoptosis/cell cycle arrest in the GBM neoplastic cells *in vitro* with clinically achievable dose of vismodegib, irrespective of their relative endogenous levels of GLI1 mRNA expression. However, the induction of apoptosis by vismodegib treatment to the GBM neurospheres *in vitro* was statistically significant albeit the overall effect size was small, suggesting a further investigation in order to improve the effect of Hh-pathway inhibitor therapies in this malignancy. Our study is suggestive of this single continuous distribution pattern of GLI1 mRNA expression technically putting almost all GBM patients in a single group in terms of Hh-pathway activity. That is encouraging for therapies with Hh-pathway inhibitor drugs without a need for stratification on the basis of relative endogenous levels of Hh-pathway activity among tumors.

## Supporting Information

S1 Table Demography of the patients included in the study. S1 Table contains list of the 19 GBM patients that were included in the study.
(XLS)

S2 Table List of primers. S2 Table contains sequences of primers that were used in quantitative RT-PCR experiments.
(XLS)

## References

[1] Nusslein-Volhard C, Wieschaus E (1980) Mutations affecting segment number and polarity in Drosophila. Nature 287: 795–801. 6776413 10.1038/287795a0

[2] Amakye D, Jagani Z, Dorsch M (2013) Unraveling the therapeutic potential of the Hedgehog pathway in cancer. Nature medicine 19: 1410–1422. 10.1038/nm.3389 24202394

[3] Ng JM, Curran T (2011) The Hedgehog's tale: developing strategies for targeting cancer. Nature reviews 11: 493–501. 10.1038/nrc3079 21614026 PMC3576812

[4] Rubin LL, de Sauvage FJ (2006) Targeting the Hedgehog pathway in cancer. Nat Rev Drug Discov 5: 1026–1033. 17139287 10.1038/nrd2086

[5] McMahon AP, Ingham PW, Tabin CJ (2003) Developmental roles and clinical significance of hedgehog signaling. Current topics in developmental biology 53: 1–114. 12509125 10.1016/s0070-2153(03)53002-2

[6] Hahn H, Wicking C, Zaphiropoulous PG, Gailani MR, Shanley S, et al (1996) Mutations of the human homolog of Drosophila patched in the nevoid basal cell carcinoma syndrome. Cell 85: 841–851. 8681379 10.1016/s0092-8674(00)81268-4

[7] Gorlin RJ, Goltz RW (1960) Multiple nevoid basal-cell epithelioma, jaw cysts and bifid rib. A syndrome. The New England journal of medicine 262: 908–912. 13851319 10.1056/NEJM196005052621803

[8] Gorlin RJ (1995) Nevoid basal cell carcinoma syndrome. Dermatologic clinics 13: 113–125. 7712637

[9] Rahnama F, Toftgard R, Zaphiropoulos PG (2004) Distinct roles of PTCH2 splice variants in Hedgehog signalling. The Biochemical journal 378: 325–334. 14613484 10.1042/BJ20031200 PMC1223965

[10] Zaphiropoulos PG, Unden AB, Rahnama F, Hollingsworth RE, Toftgard R (1999) PTCH2, a novel human patched gene, undergoing alternative splicing and up-regulated in basal cell carcinomas. Cancer research 59: 787–792. 10029063

[11] Scales SJ, de Sauvage FJ (2009) Mechanisms of Hedgehog pathway activation in cancer and implications for therapy. Trends in pharmacological sciences 30: 303–312. 10.1016/j.tips.2009.03.007 19443052

[12] Li X, Deng W, Nail CD, Bailey SK, Kraus MH, et al (2006) Snail induction is an early response to Gli1 that determines the efficiency of epithelial transformation. Oncogene 25: 609–621. 16158046 10.1038/sj.onc.1209077 PMC1361531

[13] Ruiz i Altaba A, Mas C, Stecca B (2007) The Gli code: an information nexus regulating cell fate, stemness and cancer. Trends in cell biology 17: 438–447. 17845852 10.1016/j.tcb.2007.06.007 PMC2601665

[14] Gailani MR, Stahle-Backdahl M, Leffell DJ, Glynn M, Zaphiropoulos PG, et al (1996) The role of the human homologue of Drosophila patched in sporadic basal cell carcinomas. Nature genetics 14: 78–81. 8782823 10.1038/ng0996-78

[15] Raffel C, Jenkins RB, Frederick L, Hebrink D, Alderete B, et al (1997) Sporadic medulloblastomas contain PTCH mutations. Cancer research 57: 842–845. 9041183

[16] Yauch RL, Gould SE, Scales SJ, Tang T, Tian H, et al (2008) A paracrine requirement for hedgehog signalling in cancer. Nature 455: 406–410. 10.1038/nature07275 18754008

[17] Low JA, de Sauvage FJ (2010) Clinical experience with Hedgehog pathway inhibitors. J Clin Oncol 28: 5321–5326. 10.1200/JCO.2010.27.9943 21041712

[18] Rudin CM (2012) Vismodegib. Clin Cancer Res 18: 3218–3222. 10.1158/1078-0432.CCR-12-0568 22679179 PMC3715061

[19] Rudin CM, Hann CL, Laterra J, Yauch RL, Callahan CA, et al (2009) Treatment of medulloblastoma with hedgehog pathway inhibitor GDC-0449. The New England journal of medicine 361: 1173–1178. 10.1056/NEJMoa0902903 19726761 PMC5317279

[20] Berlin J, Bendell JC, Hart LL, Firdaus I, Gore I, et al (2013) A randomized phase II trial of vismodegib versus placebo with FOLFOX or FOLFIRI and bevacizumab in patients with previously untreated metastatic colorectal cancer. Clin Cancer Res 19: 258–267. 10.1158/1078-0432.CCR-12-1800 23082002

[21] Kaye SB, Fehrenbacher L, Holloway R, Amit A, Karlan B, et al (2012) A phase II, randomized, placebo-controlled study of vismodegib as maintenance therapy in patients with ovarian cancer in second or third complete remission. Clin Cancer Res 18: 6509–6518. 10.1158/1078-0432.CCR-12-1796 23032746

[22] Stupp R, Mason WP, van den Bent MJ, Weller M, Fisher B, et al (2005) Radiotherapy plus concomitant and adjuvant temozolomide for glioblastoma. The New England journal of medicine 352: 987–996. 15758009 10.1056/NEJMoa043330

[23] Dahmane N, Sanchez P, Gitton Y, Palma V, Sun T, et al (2001) The Sonic Hedgehog-Gli pathway regulates dorsal brain growth and tumorigenesis. Development (Cambridge, England) 128: 5201–5212. 11748155 10.1242/dev.128.24.5201

[24] Hu Z, Bonifas JM, Aragon G, Kopelovich L, Liang Y, et al (2003) Evidence for lack of enhanced hedgehog target gene expression in common extracutaneous tumors. Cancer research 63: 923–928. 12615704

[25] Bar EE, Chaudhry A, Lin A, Fan X, Schreck K, et al (2007) Cyclopamine-mediated hedgehog pathway inhibition depletes stem-like cancer cells in glioblastoma. Stem cells (Dayton, Ohio) 25: 2524–2533. 17628016 10.1634/stemcells.2007-0166 PMC2610257

[26] Gruber Filbin M, Dabral SK, Pazyra-Murphy MF, Ramkissoon S, Kung AL, et al (2013) Coordinate activation of Shh and PI3K signaling in PTEN-deficient glioblastoma: new therapeutic opportunities. Nature medicine 19: 1518–1523. 10.1038/nm.3328 24076665 PMC3923315

[27] Brennan CW, Verhaak RG, McKenna A, Campos B, Noushmehr H, et al (2013) The somatic genomic landscape of glioblastoma. Cell 155: 462–477. 10.1016/j.cell.2013.09.034 24120142 PMC3910500

[28] Johnson RL, Rothman AL, Xie J, Goodrich LV, Bare JW, et al (1996) Human homolog of patched, a candidate gene for the basal cell nevus syndrome. Science (New York, NY 272: 1668–1671. 8658145 10.1126/science.272.5268.1668

